# Administration of adalimumab in paediatric patients with juvenile idiopathic arthritis in South Ural region

**DOI:** 10.1186/1546-0096-12-S1-P340

**Published:** 2014-09-17

**Authors:** Galina Glazyrina

**Affiliations:** 1 Hospital Pediatrics, South Ural State Medical University, Chelyabinsk, Russian Federation

## Introduction

Juvenile idiopathic arthritis (JIA) is a severe disabling disease affecting paediatric population. 507 children with JIA are monitored in Chelyabinsk region. Oligo-articular and poly-articular types of JIA are often associated with affection of eyes (uveitis). In patients resistant to traditional basic treatment (methotrexate and cyclosporine) F-alpha inhibitors are recommended. Adalimumab, approved for treatment of children older than 4 years in the Russian Federation, is a treatment of choice in patients with JIA associated with uveitis.

## Objectives

Evaluate adalimumab effectiveness and safety in JIA patients from Ural regions, Russia

## Methods

11 JIA patients (age 8-17 years, mean age 12.2 years, 7 M, 4 F) were monitored. Disease duration: from 1 year to 11 years (mean 7.4 years). JIA diagnosis was based on the ILAR criteria. Oligo-arthritis was reported in 5 children, seronegative polyarthritis was reported in 4 children. Systemic (without active systemic manifestations) and enthesitis – associated JIA types were reported in 1 patient each. 7 children had uveitis (bilateral uveitis was reported in 6 of them). High disease activity was reported in 7 patients. X-ray activity (stages 1-2) was reported in 8 children. All patients had functional class 2 disease. Methotrexate (15 mg/m^2^) was ineffective in all children. All patients were receiving adalimumab (40 mg s/c EOW). Treatment course duration: from 3 months to 3.5 years (mean 1.3 years). ACR pedi criteria were used to evaluate the disease activity and treatment efficacy.

## Results

High disease activity (both joint syndrome and uveitis) was reported in all children prior to adalimumab initiation. Mean number of joints affected by severe arthritis: 5 [3, 8] ([25; 75%]). Number of joints with dysfunction: 4 [3, 8]. Mean ESR (as measured by Panchenkov’s method): 20 [15, 32] mm/hour; C-RP level: 25 [13, 40] g/l. Bilateral uveitis was reported in 6 patients; unilateral uveitis was reported in 1 patient. Number of affected eyes: 13. Uveitis was mild and associated with cataract, resulting in lens replacement in 11 affected eyes. Adalimumab treatment resulted in disease activity decrease in all patients.

Mean number of joints affected by active arthritis was 0 [0, 3] ([25; 75%]) (P = 0.003). Number of joints with function disorder – 0 [0, 3] (P = 0.003). Mean ESR level: 4 [2, 15] mm/hour (P = 0.005), C-RP level: 6.5 [3; 15] g/l (P = 0.008). CHAQ function index: 0.19 [0; 0.5] (P = 0.003). Investigator's assessment of disease activity (VAS): 20 [15, 30] (P=0.003). Patients/parents assessment of disease activity (VAS): 20 [20, 30] (P = 0.003). Clinical remission (ACR pedi criteria ≥90%) was reported in 8 patients within 3-6-12 months of treatment. 2 patients were lost for contact within 3 and 6 months of treatment; last reported efficacy was 50%. Insufficient efficacy was reported only in 1 patient with systemic JIA (50% within 2.5 treatment years), resulting in treatment regimen change. Remission of uveitis within 3 months of treatment was reported in 2 children; remission within 6 months of treatment was reported in 3 children. Significant decrease of uveitis activity was reported in 2 children.

Adverse event (transient myositis) was reported in 1 patient; treatment withdrawal was not required.

## Conclusion

In patients with JIA, including uveitis-associated cases, adalimumab treatment was highly effective and safe. Clinical remission was achieved in 70% of children. Disease activity decrease was reported in 100% of patients. No severe adverse events were reported.

## Disclosure of interest

None declared.

